# The National Medical Societies

**Published:** 1894-07

**Authors:** 


BUFFALO MEDICAL AND SURGICAL JOURNAL

A MONTHLY REVIEW OF MEDICINE AND SURGERY.

*EDITORS:*

THOMAS LOTHROP, M. D. - - WM. WARREN POTTER, M. D.

All communications, whether of a literary or business nature, should be addressed to the managing editor: 284 FRANKLIN STREET, BUFFALO, N. Y.

VOL. XXXIII. JULY, 1894. No. 12.

### THE NATIONAL MEDICAL SOCIETIES.

THE Spring meetings for the year 1894 have been held, and the work of the societies has passed into history. The medical journals are publishing the papers, and will continue to do so until all have appeared, which, probably, will occupy most of the available space for the remainder of the year.

Whether the work accomplished will constitute a contribution to medical literature that will advance the science and art of medicine, remains to be determined when the record shall be completed and the transactions published. Some idea, however, as to the quality of the work done has been foreshadowed by the synopsis published in the several medical weeklies.

The Congress of American Physicians and Surgeons, that embraces several of the special associations, met in Washington, May 29, 30, 31 and June 1, 1894, and gathered together something over 400 of the representative specialists of the country, and this organization may properly be designated as the medical "Four Hundred." It may be expected that the work done in Washington will prove, for the most part, of a scientific nature, yet there must be necessarily some chaff even among the best quality of wheat, but the separator—the medical journals of the land—will soon get in its work.

This Congress has one great essential feature of commendation—namely, that it devotes its entire time to the consideration of scientific papers, and does not allow medical politics to interfere with its work. It has no code of ethics to wrangle over, and yet its members are gentlemen! Its executive committee performs the entire business part of its work, even to the choosing of its officers and to designating its subordinate committees. It permits no sideshows to attract its members, and its model is worthy of imitation.

Let us turn to the other side of the picture. The American Medical Association goes junketing across the continent and appoints its meeting in San Francisco, in the year of the greatest financial distress that the country has experienced since antebellum days. Ordinarily speaking, from a business standpoint it would be considered poor policy to appoint a meeting at such a distance in such a year. But one need not go so far in search of the real object without finding it. Last year, at Milwaukee, it found itself confronted with constitution and code amendments that its old masters feared to meet in the East, where the majority has been steadily growing against them.

If there is anything the Nestors of the American Medical Association dread, it is a change in its code of ethics. These men cannot recognize or contribute to the progress that is everywhere around them taking place. So it seeks to dodge the question by appointing the meeting in San Francisco, in the expectation that few from the East would attend, and, as if to make this more certain, the date was fixed so near the meeting of the Washington Congress as to practically prevent attendance upon both. At San Francisco, a majority of the committee was in favor of revision, and so, as it proved, was a majority of its members. This was shown by the fact that 151 votes were cast in favor of, and 64 against, revision, but the astute chairman, one of the old hardshells of the Association, announced that a three-fourths vote was required to change the constitution ; so the proposition was defeated, hence everything stands as before the San Francisco meeting.

The aggregate attendance was about 600, a large proportion of which came from the Pacific slope. Some of the section meetings were poorly attended, necessitating the consolidation of a few, and the work of the Association, as a whole, fell below the average standard.

Dr. Donald Maclean, of Detroit, was chosen president, and the next meeting was appointed to be held in Baltimore, beginning on the first Tuesday of May, 1895, with Dr. Julian J. Chisholm, of Maryland, as chairman of the committee of arrangements. Let us hope that the Baltimore meeting will result in a work of redemption for the American Medical Association.

